# A Review of Refractometric Sensors Based on Long Period Fibre Gratings

**DOI:** 10.1155/2013/913418

**Published:** 2013-12-18

**Authors:** G. Rego

**Affiliations:** ^1^Escola Superior de Tecnologia e Gestão, Instituto Politécnico de Viana do Castelo, Avenida do Atlântico, 4900-348 Viana do Castelo, Portugal; ^2^UOSE, INESC-Porto, Rua do Campo Alegre 687, 4169-007 Porto, Portugal

## Abstract

In the last decade refractometric sensors have attracted an increasing interest by the scientific community due to their ability to perform ambient monitoring, to assess food quality and safety, and also to the fact that they enable the development of label free sensors in the biomedical area. These advances result, namely, from the use of long period fibre gratings in the turning points and/or with thin films in the transition region that allows resolutions of 10^−6^ to changes in the refractive index of the surrounding medium. Resolutions exceeding 10^−8^ can also be achieved when long period fibre gratings are combined with evanescent field based devices. This paper reviews the recent path towards the development of ultrahigh sensitive optical fibre refractometric sensors.

## 1. Introduction

According to recent designations such as smart cities, smart buildings, smart structures, smart grids, or smart clothes, one may conclude that we are living in the “Smart World Era.” Generally speaking, these environments require intelligent devices that incorporate sensors, communication systems, and data processing unities that are able to perform home automation, to monitor structural health of buildings and bridges, to increase the efficiency of energy production, and to manage an electric power grid or to localize patients and monitor their vital parameters. Further developments are expected with the so-called “Internet of Things” in which intelligence is extended to, virtually, all objects [1, 2].

At the same time the world faces the challenge caused by the destructive cycle of environmental pollution, global warming, climate changes, reduction of arable land, and water contamination, added to the demographic pressure and a huge ecological footprint that demands for another planet. Furthermore, the occidental society has a higher life expectancy and diseases, such as, Alzheimer, which are more frequent. Therefore, the future development requires special attention to these issues and the smart technology plays an important role in order to increase the efficiency of food production and to control its safety, to monitor the quality of air and water, and to localize and monitor the health of elder people [3].

In this context, sensors are fundamental devices independently of being the conventional electronic ones (that may constitute a wireless sensor network when, for instance, mobility is required) or optical fibre sensors used in applications where low dimensions, low weight, electromagnetic immunity, and material chemically inert and biocompatible are essential. Since the optical fibre can be, simultaneously, a waveguide and a sensor, it is straightforward to implement optical fibre sensor networks that enable multiplexing of sensors monitoring different physical parameters. As far as the measurement of temperature and strain is concerned, for instance, for health structure monitoring, the fibre Bragg gratings technology has already proved its excellence [4]. Distributed sensing based on the Brillouin effect is paving the way towards the development of temperature sensors, for instance, in the area of energy production. However, in applications such as environmental monitoring or food quality and safety, other approaches are needed. Refractometric sensors based on long period fibre gratings (LPFGs) have shown very promising results in this context [5]. Therefore, this paper reviews the recent achievements related to this technology.

The first section discusses the fabrication of long period fibre gratings in the turning points using the simple, flexible, and low cost electric arc technique. Based on the knowledge of the properties of these gratings a reflection configuration is also presented. The deposition of thin films over the grating, namely, when the film has a particular thickness and refractive index that enables the grating to work in the transition region increasing considerably the sensitivity to changes in the external refractive index, is discussed in Section 2.  Section 3 present other configurations for refractometric sensing based on evanescent field that may combine different devices in order to reach ultimate resolutions, for instance, schemes comprising long period fibre gratings and surface plasmon resonances (SPR).

## 2. Properties of Arc-Induced Gratings

**Figure 1:**
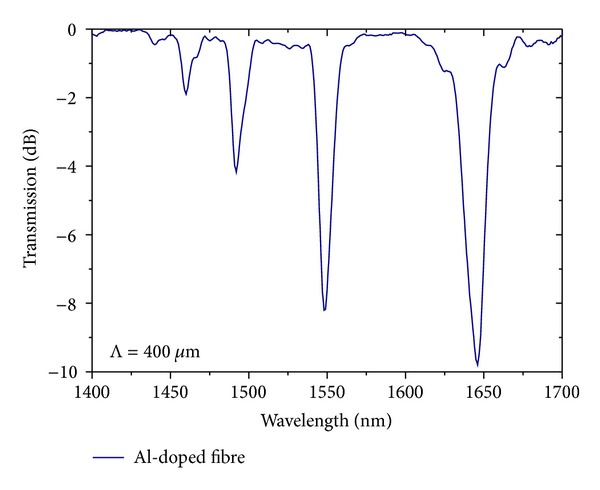
Typical spectrum of a 400 *μ*m-LPFG inscribed in an Al-doped fibre.

A long period fibre grating is a wavelength selective filter whose transmission spectra exhibit several resonances (Figure 1) resulting from coupling between the core mode and the different copropagating cladding modes at wavelengths that obey the resonance condition [6]:

(1) $$\begin{array}{c} \lambda_{\text{res}}=\left(n_{\mathrm{co}}^{\text{eff}}-n_{\mathrm{cl},m}^{\text{eff}}\right)\Lambda , \end{array}$$

where *λ*_res_ represent the resonance wavelengths, Λ the grating period, and *n*_co⁡_^eff^ and *n*_cl⁡,*m*_^eff^ the effective refractive index of the core mode and the effective refractive index of the cladding modes, respectively.

Several techniques are available in order to produce LPFGs, among them, those based on UV, CO2, and femtosecond laser radiation, with the later being the most versatile one [6–9]. However, we focused our attention on the electric arc technique for being simple, flexible and having low cost [10]. The main drawback is the fact that so far it was not possible to write LPFGs with grating periods shorter than 200 *μ*m [11]. Arc-induced gratings are fabricated by placing an uncoated fibre, under tension, between the electrodes of a splicing machine. The fibre is then submitted to an arc discharge with an electric current of 7 to 15 mA and a duration ranging from 200 ms up to 2 s. Afterwards the fibre is displaced by the grating period, typically, from 200 *μ*m to 1 mm and the whole process of arc-discharge/fibre displacement is then repeated 20 to 50 times.

The reproducibility of the technique can be compromised by the degradation of the electrodes due to oxidation. However, the control of the position of the fibre in the arc discharge and the monitoring of the discharge parameters and of the ambient conditions would allow the technique to be used for mass production of gratings with precise and reproducible characteristics [12].

**Figure 2:**
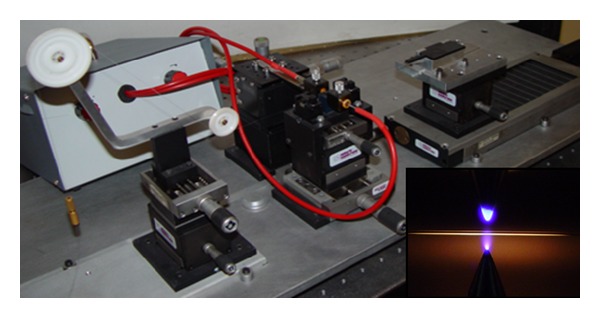
Setup for the fabrication of arc-induced gratings.

Figure 2 shows the setup used to inscribe the gratings where it can also be seen that the arc discharge is directional. It was demonstrated that, in this particular arrangement, the arc possesses a thermal gradient that is responsible for the main formation mechanism of these gratings in standard fibres, that is, microdeformations [12]. For the typical fabrication parameters used the fibre reaches a temperature of the order of 1400°C [13] which softens the fibre under tension and the arc thermal gradient induces the microdeformations responsible for the grating. Arc-induced gratings can stand temperatures as high as 1000°C for several days without significant deterioration in their spectrum [14]. It was also demonstrated that the resonance wavelengths are defined not only by the choice of the grating period but also by the fabrication parameters used [15]. Moreover, the fabrication parameters also modify the sensitivity of the resonances to changes of physical parameters such as temperature and strain. Thus, by changing the fabrication parameters during the inscription of a grating we obtained a set of two neighbour resonances showing different sensitivities that enabled to implement a sensor for the simultaneous measurement of temperature and strain requiring a single optical source [16]. It is interesting to note that in the case of the fibre codoped with B/Ge the main mechanism of grating formation is not microdeformations but a reversible densification of the core that leads to coupling to symmetric cladding modes in opposition to the asymmetric ones due to the former mechanism [17, 18]. However, by a proper choice of the fabrication parameters and by placing the fibre in a region of the arc with lower average temperature and grater thermal gradient (i.e., also an optimum point to increase the reproducibility of the technique) it is possible to fabricate simultaneously two superimposed gratings showing a dual set of resonances resulting from two different mechanisms: microdeformations and densification [19]. The resonances show different sensitivities to changes in physical parameters and the ones due to densification are erased as the temperatures rise towards 1000 °C. The development of sensors based on these properties will be discussed below.

The resonance wavelengths depend on the effective refractive index of the cladding modes which in turn depend on the refractive index of the external medium. Thus, LPFGs are intrinsically sensitive to changes in the surrounding medium and, therefore, can be used as refractive index sensors. Several techniques have been used in order to increase the gratings sensitivity to changes in the external refractive index that depends on the interaction of the cladding modes with the surrounding medium. For that purpose several approaches were implemented: gratings were written in fibres having a cladding refractive index closer to the one of the surrounding medium, written in etched or tapered fibres, by producing microtapered gratings or by bending the grating. In this context, interferometric configurations were also used. The resolutions obtained ranged from 10^−3^ to 10^−5^ [20–26].

## 3. LPFGS in the Turning Points

**Figure 3:**
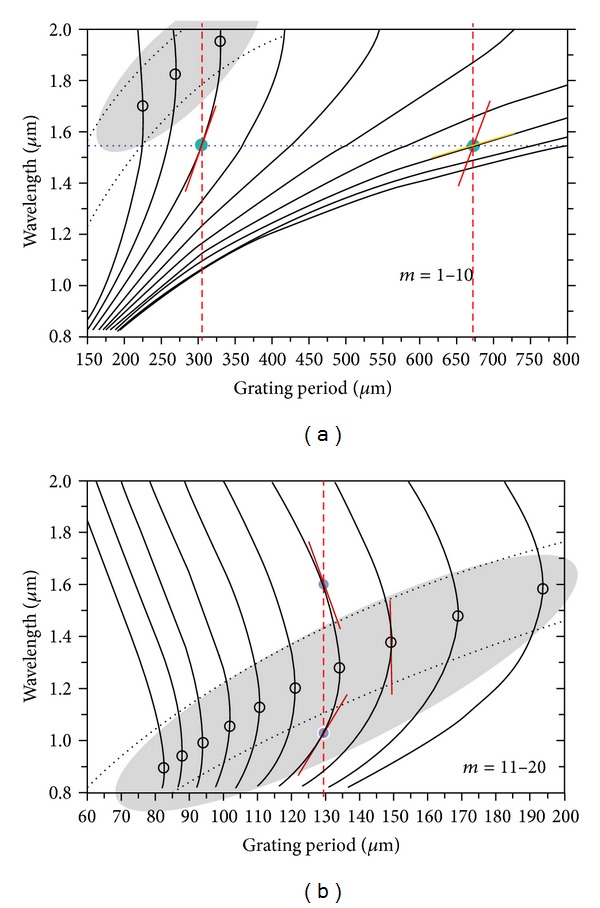
Phase matching curves. LPFGs turning points (adapted from [27]).

It is well known that the LPFGs sensitivity depends on the order of the cladding modes and reaches its maximum close to the so-called turning points [27]. In these regions the slope of the phase matching curves changes from positive to negative and beyond this turning point, and for each grating period, there are two resonance wavelengths for each cladding mode (Figure 3). This is due to the dependence on wavelength of the core and cladding effective refractive indices. For a particular grating period, the phase matching condition can be satisfied for more than one resonance wavelength (for the same cladding mode) since, as the wavelength increases, the effective refractive index of the cladding mode decreases faster than that of the core [28].

In the context of refractometric sensing, the dependence of the resonant wavelengths on changes in the refractive index of the surrounding medium can be understood by the following equations [27]:

(2) $$\begin{array}{c} \frac{d\lambda_{\text{res}}}{dn_{\text{surr}}}=\lambda_{\text{res}}\gamma \Gamma_{\text{surr}}, \end{array}$$

where *γ*, the general sensitivity factor, describes the waveguide dispersion and is expressed by

(3) $$\begin{array}{c} \gamma =\frac{d\lambda_{\text{res}}/d\Lambda }{n_{\mathrm{co}}^{\text{eff}}-n_{\mathrm{cl},m}^{\text{eff}}}, \end{array}$$

where *dλ*_res_/*d*Λ represents the slope of the dispersion curves. Γ_surr_ is expressed by

(4) Γsurr=−um2λres3nsurr8πrcl⁡3ncl⁡(nco⁡eff−ncl⁡,meff)(ncl⁡2−nsurr2)3/2             (valid  for  nsurr<ncl⁡)

and describes the dependence of the waveguide dispersion on the surrounding refractive index (*n*_surr_). The term *u*_*m*_ is the *m*th root of the zero-order Bessel function and the other symbols have their common meaning. From (2) and (3) it can be concluded that the sensitivity increases with the slope of the dispersion curves (requires higher order cladding modes and/or working near the turning points). Note that for a particular mode the resonance wavelengths beyond the turning points show higher sensitivities (see also Figure 3). Equation (4) also shows that the sensitivity increases as the refractive index of the surrounding approaches that of the cladding. In this case the evanescent field extends further into the surrounding medium leading to a higher interaction. However, when the surrounding refractive index equals that of the cladding (it becomes an infinite medium), the resonances disappear. For refractive indices above that of the cladding the resonances reappear but at longer wavelengths [29]. In this case, the modes are guided by Fresnel reflection and not by total internal reflection (when *n*_surr_ > *n*_cl⁡_, there is no critical angle) and loose part of the energy at each reflection at the interface being called lossy modes. As the refractive index increases the modes become more confined and the dips more pronounced. However, the resonance wavelengths are insensitive to changes in the surrounding refractive index because the phase of the partially reflected field at the surrounding-cladding interface does not change with the external index [30, 31].

**Figure 4:**
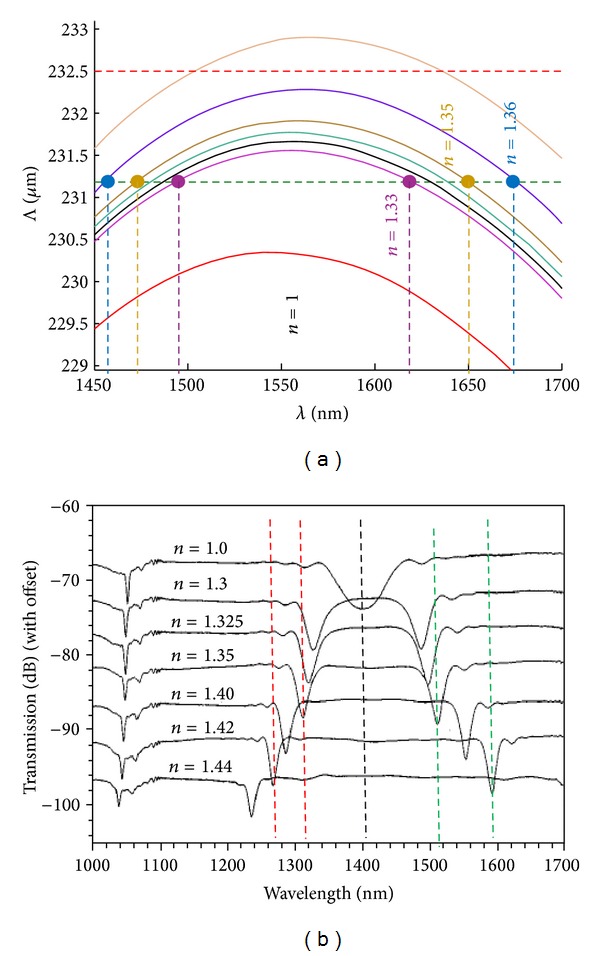
(a) Phase matching curve of a particular mode as a function of the external refractive index. (b) Displacement of the resonance wavelengths for different refractive indices (adapted from [27, 33]).

In general, grating periods shorter than 200 *μ*m are required in order to have access to this sensitive region (around the turning points), but this possibility was not yet demonstrated for arc-induced gratings. Presently we are developing a new power supply to produce a stable arc with reduced dimensions. At the same time, it is known that during an arc discharge the fibre reaches thermal equilibrium in less than half a second [32] and, therefore, a shorter arc duration will mitigate the effect of thermal diffusion. Thus, the combination of these two factors will allow us to fabricate arc-induced gratings in the turning points. Depending on the choice of the grating period around the turning point, the changes on the surrounding refractive index can be monitored by following either the wavelength shift or the intensity variation [27, 33] (see Figure 4). Recently, two sensors based on LPFGs in the turning points (intensity/wavelength) showed a resolution of 3-4 × 10^−6^ [34, 35].

**Figure 5:**
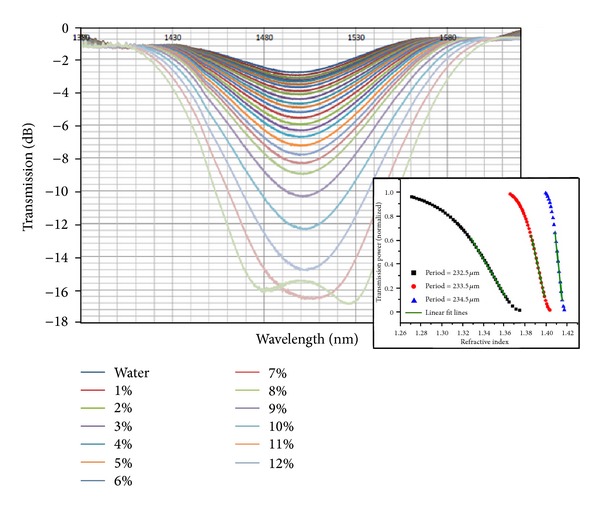
Change of the intensity of a particular mode as a function of the external refractive index. Inset: the choice of the period enables the tuning of the sensitivity for different refractive indices to be measured (taken from [33, 34]).

In the case of the wavelength measurement a *π*-shifted LPFG in the turning points was used. It should be stressed that a proper choice of the grating period allows some tuning of the sensitivity for a particular refractive index range to be measured (see Figure 5). Further tuning can also be accomplished by etching the fibre cladding [36].

**Figure 6:**
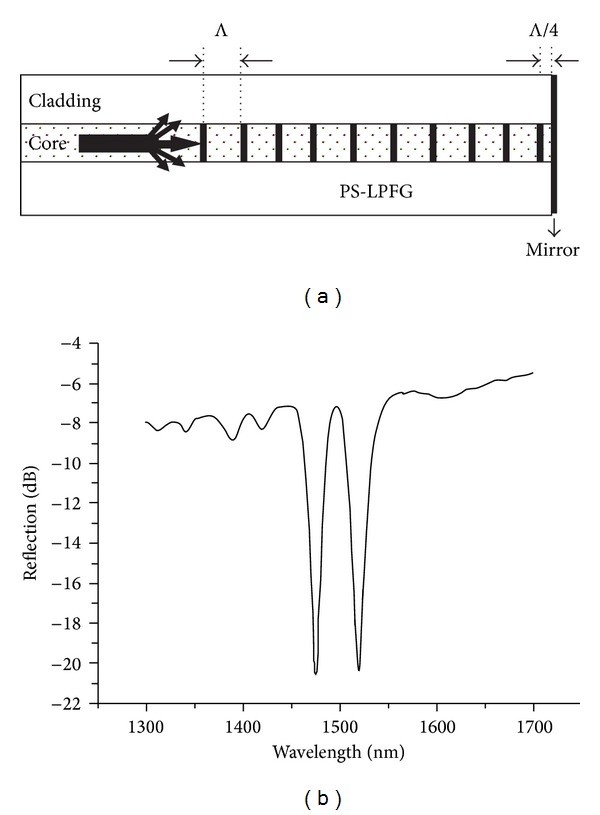
(a) Reflection configuration based on a *π*-shifted LPFG; (b) spectrum of the bandpass filter (taken from [32]).

**Figure 7:**
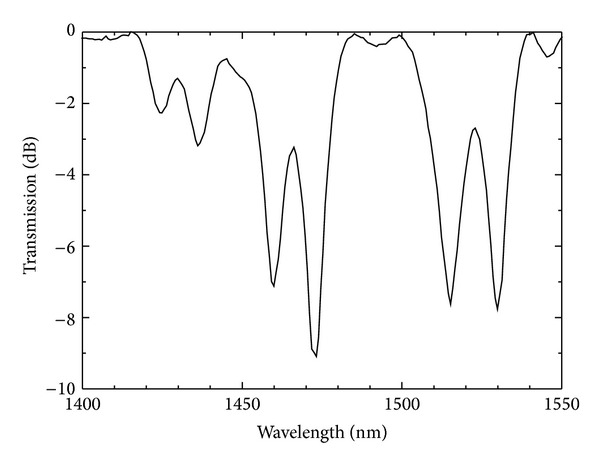
Gratings spectrum with a dual set of resonances (Λ = 540 *μ*m).

A drawback of using sensors based on LPFGs is the fact that they need access to both ends of the fibre. In a recent publication [37], the authors proposed the inscription of a *π*-shifted LPFG [38, 39] by the deposition of a mirror at the end of the fibre at a distance of Λ/4 to the grating (Figure 6). In this way they implemented a sensor working in a reflection configuration for the simultaneous measurement of temperature and refractive index. The difference in the sensitivity obtained for both resonances is small due to the proximity of the resonance wavelengths. However the sensitivities can be increased considerably if one inscribes simultaneously two gratings, near the turning points, exhibiting a dual set of resonances with different symmetries in the B/Ge-doped fibre (Figure 7) [19]. In this way we end up with a sensor head having the advantage of working in reflection, plus the higher sensitivity of the turning points and the distinct sensitivities of the resonances belonging to two *π*-shifted LPFGs of different symmetries. This configuration enables the simultaneous measurements of different parameters by using either the resonance wavelengths or the resonances of the bandpass filters. This ability is important to overcome the problem of cross-sensitivity to other parameters, such as temperature. Note that the separation between the symmetric and asymmetric resonance wavelengths can be increased by using shorter grating periods. This sensor head is currently under development (at this stage the gratings were not written in the turning points) and the results will be published elsewhere.

## 4. Thin Film-Coated LPFGs

**Figure 8:**
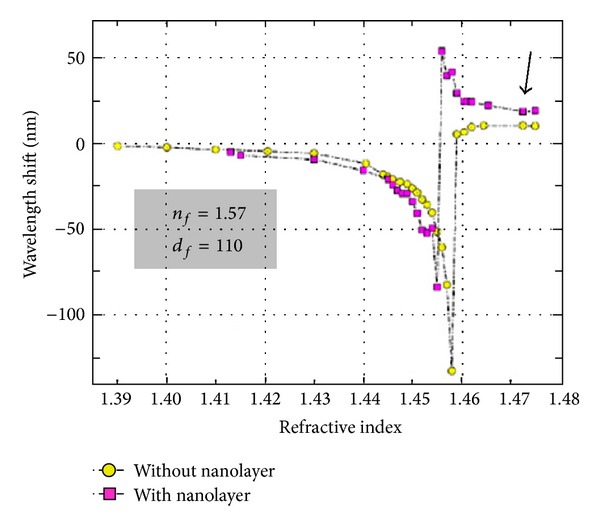
Thin film-coated LPFG for assessing the quality of fried oil (taken from [42]).

Although the inscription of LPFGs in the turning points increases their sensitivity to changes in the surrounding refractive index, it is known that the sensitivity is higher as the refractive index of the surrounding approaches that of the cladding. This imposes a limitation to work with gases or water solutions. Furthermore, gratings are also insensitive to refractive indices above that of the cladding. Therefore, means to overcome those limitations were required. In 2002, Rees et al. [40] demonstrated that the deposition of a thin film over the grating, with a refractive index exceeding that of the cladding, changes the position of the resonances. As the film thickness increases, the average external refractive index also increases leading to a down shift of the resonances. A point is reached where the resonances disappear. This was attributed to the fact that the average index of the external medium equals that of the cladding. With further deposition the resonances reappear but at a higher wavelength followed by a down shift towards the values obtained for the case of a LPFG surrounded by an infinite medium. The deposition of a thin film over the grating slightly increased the sensitivity to changes in the refractive index of the surrounding medium lower than that of the cladding. On the other hand, it allowed the measurement of refractive index changes of the surrounding medium with a refractive index above that of the cladding [41]. For instance, the deposition of a 110 nm thin film with a refractive index of 1.57 allowed assessing the quality of fried oil with a refractive index of ~1.47 (Figure 8) [42].

**Figure 9:**
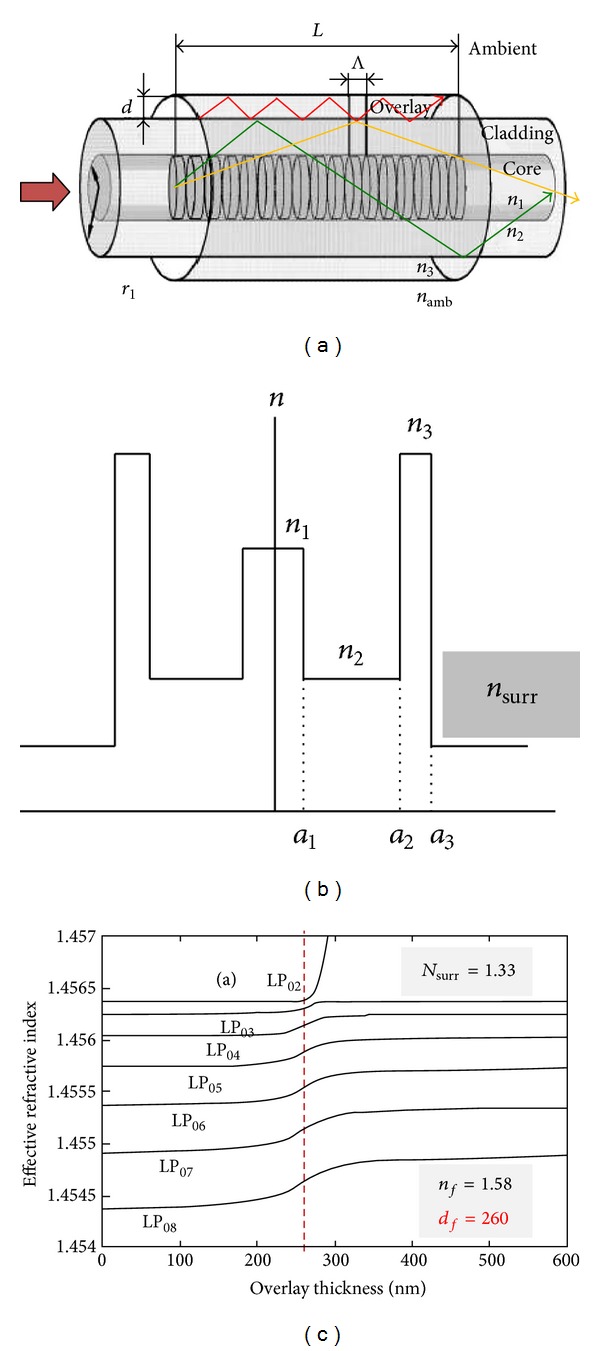
(a) A mode guided by the thin film (the ray trace in red), (b) refractive index profile, and (c) the transition region (adapted from [43, 44]).

Subsequent research [43–46] enabled a deeper interpretation of the transition region, that is, the region where the resonances of LPFGs coated with the Langmuir-Blodgett technique disappear. In fact, with the increase of the film thickness a cladding mode is allowed to be guided in the film leading to a rearrangement of the cladding modes in which a cladding mode order will replace the position of the cladding mode with the precedent order (e.g., LP_08_ replaces LP_07_; see Figure 9). This is a fast transition leading to the highest sensitivity.

**Figure 10:**
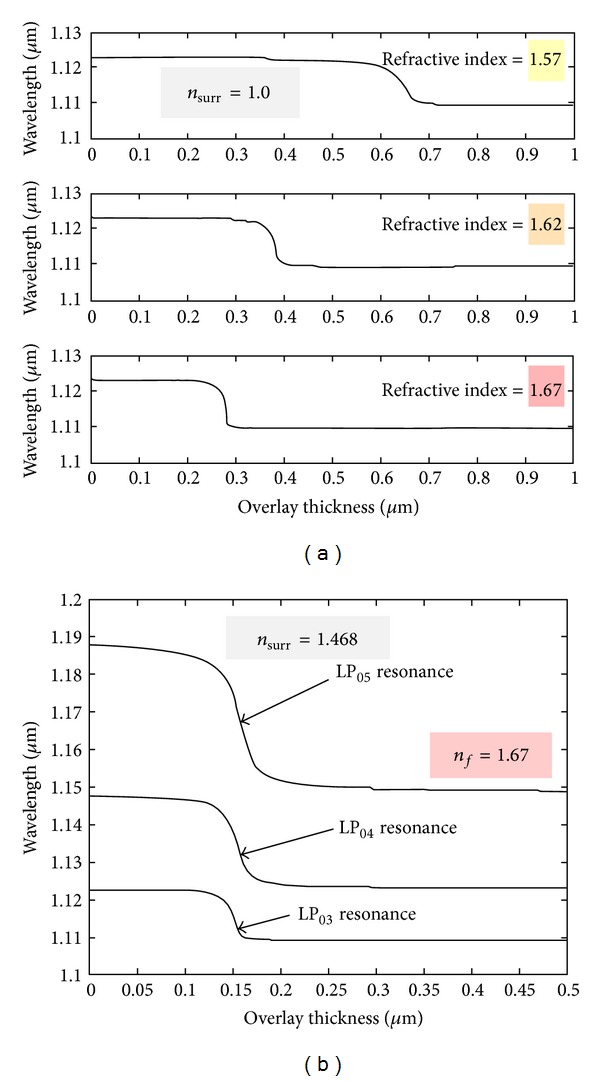
The transition region. (a) Dependence of the optimum thickness on the refractive index of the thin film and (b) on the surrounding refractive index (taken from [46]).

The higher the film refractive index, the lower the thickness required to reach the transition region (see Figure 10). The thickness also decreases as the surrounding refractive index approaches that of the cladding [46]. Both facts as well as higher cladding modes order lead to faster transitions (higher sensitivity).

**Figure 11:**
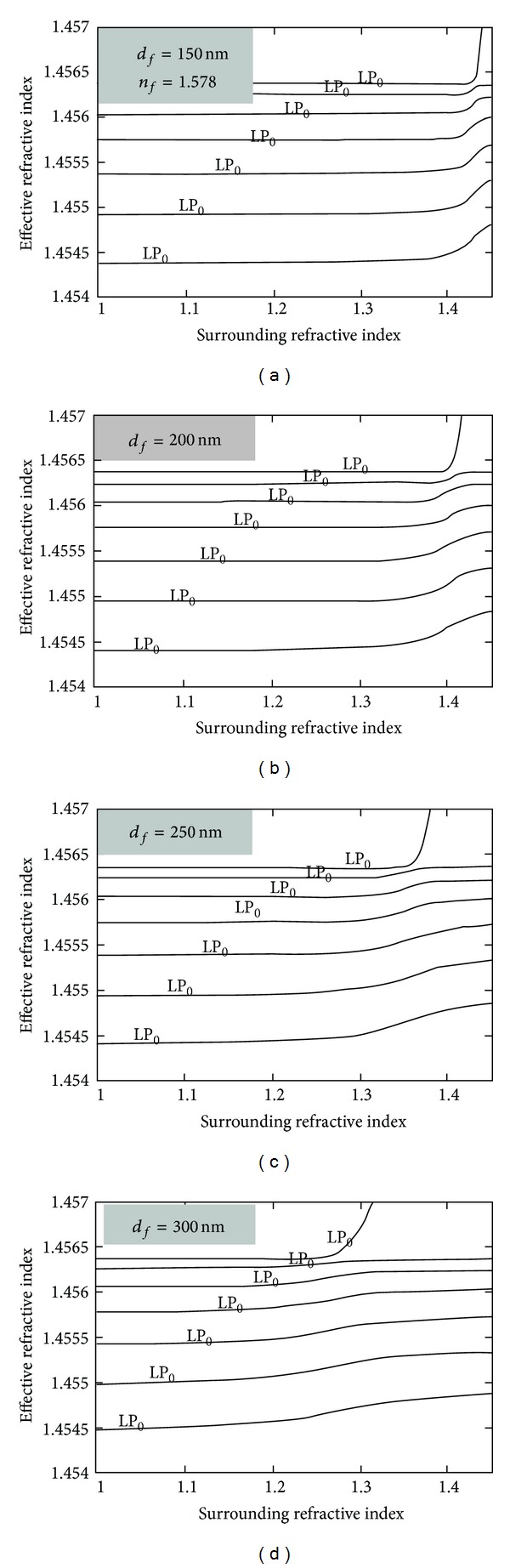
Dependence of the transition region on the film thickness (taken from [47]).

On the other hand the transition region occurs for lower values of the surrounding refractive index as the film thickness increases. This also contributes to a decrease of the sensitivity [47] (Figure 11). Therefore, a proper choice of the refractive index and thickness of the film would allow the implementation of sensors optimized for refractive indices lower than that of the cladding such as applications in air or water environments.

**Figure 12:**
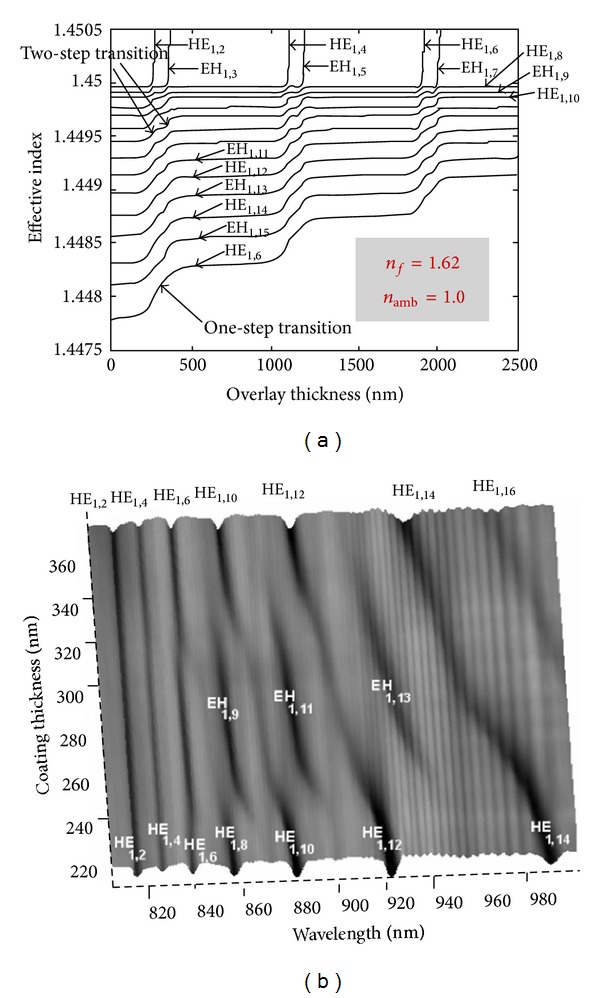
(a) The transition region. (b) Mode conversion and mode reorganization (taken from [48]).

**Figure 13:**
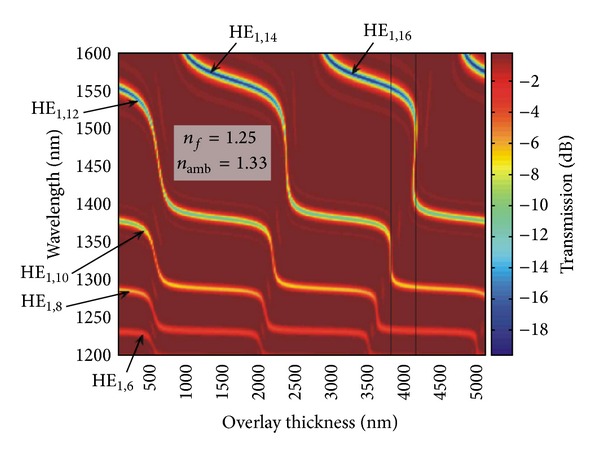
Higher order transitions (taken from [50]).

However in the optimum point, that is, a particular film thickness, the resonances almost disappear making it impossible to follow the resonance change. The reasons for that disappearing are the film loss and the fact that during the transition the modes also change from HE to EH before replacing the HE mode of lower order [48–52]. Note that for high loss films it is not always the lowest order cladding modes to be guided first by the film (Figure 12). The resonances below the one that is guided by the film behave differently from those above it.Figure 13 shows that, as the film thickness increases, other transitions will occur. Transitions are faster for higher order transitions [50].

**Figure 14:**
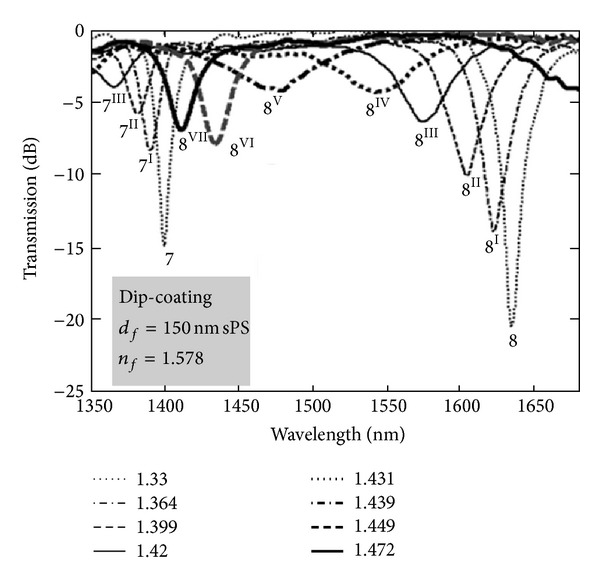
Resonances in the transition region of a dip-coated film (Taken from [47]).

Therefore care must be taken in order to stop the film deposition before the optimum point and to control the film loss by a proper choice of the deposition technique. In fact it is known that the dip-coating technique leads to films with lower loss but at the same time does not allow a good control of the thickness [5].  Figure 14 shows that, although the coupling strengths decrease in the transition region, the resonances are still visible [47]. Thus, the best option would be a combination of two techniques, first dip-coating to control loss and then electrostatic self-assembly to control the thickness [53].

**Figure 15:**
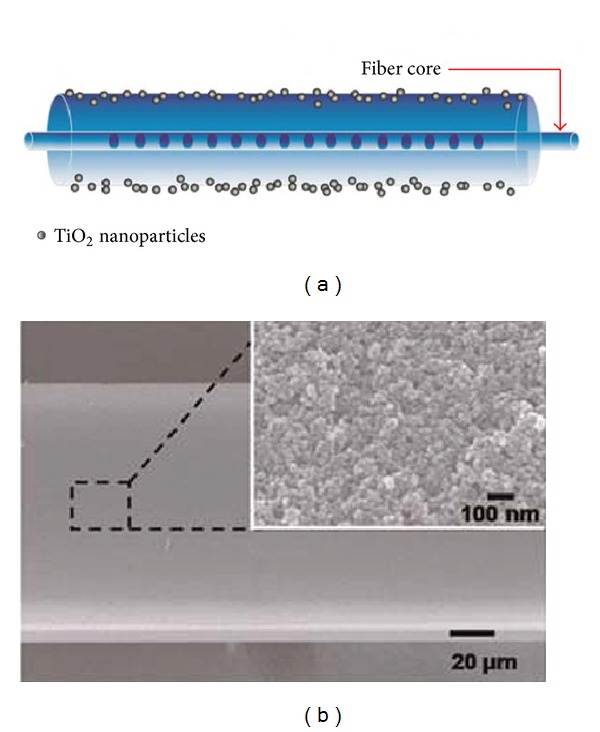
Glucose measurement using a LPFG coated with TiO_2_ nanoparticles (taken from [65]).

The deposition of two thin films in which the first layer has a smaller refractive index than the second layer forms a tunnel effect that leads to a more pronounced two step transitions [54]. On the other hand, it is possible to use a first layer to increase the overall sensitivity and a second one being selective to a particular chemical or biologic component [55]. Thin films sensitive to specific components, such as, for pH measurement or for detection of Cl^−^, chloroform, hydrogen, methane, and ethanol, have also been used [44, 56–62]. In this case, the sensitivity of the film increases if its refractive index approaches that of the cladding [56]. Finally, the combination of LPFGs in the turning points with thin films in the transition region led to the implementation of a sensor with a resolution of the order of 10^−6^@1.35 [5]. LPFGs in the turning points coated with thin films have been used for detection of ammonia, *E. coli*, and glucose [63–65]. In the latter case the properties of the film, such as its permeability to the analyte, were taken into account in order to increase the sensitivity (Figure 15). Thus, instead of a simple adsorption to the surface that mainly increases the film thickness, there is diffusion into the film leading to an overall increase on its refractive index. It is expected that future research on these subjects will contribute to a further increase in the sensitivity of refractometric sensors.

## 5. LPFGs-Assisted SPR

The sensitivity of refractometric sensors depends strongly on the interaction between the evanescent field and the analyte. Therefore, it can be increased by reducing the fibre diameter. Tapering the fibre to dimensions of a few micrometers or even to nanometers scale leads to resolutions of 10^−6^–10^−7^ RIU [66–68]. The interaction can also be increased by introducing the analyte in the holes of a PCF fibre leading to similar resolutions [69–71]. Another remarkable approach is based on the coupling to whispering gallery modes (WGM) of a resonator [72]. The combination of nanotapers and microresonators led to the detection of a single virus [73].

**Figure 16:**
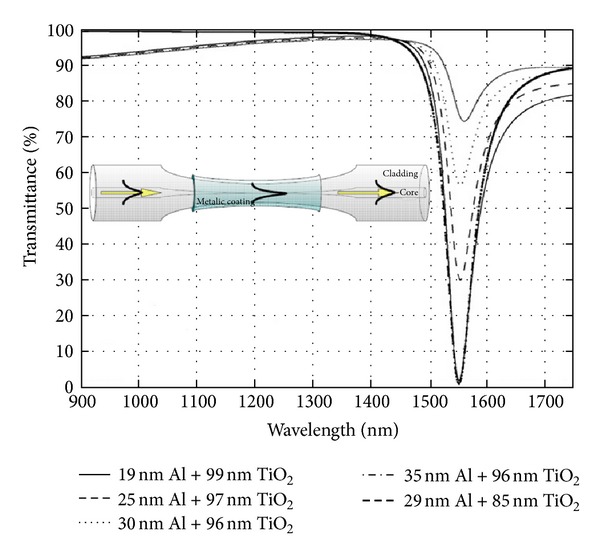
Fibre taper SPR, with different film thickness, showing resonances in the third telecommunication window (taken from [79]).

**Figure 17:**
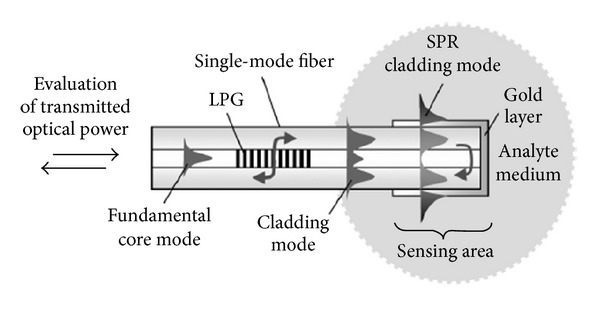
Refractometric sensor in a reflection configuration based on a LPFG-assisted SPR (taken from [82]).

It is well known that the refractometric sensors based on surface plasmon resonance (SPR) have demonstrated the highest sensitivity, although for bulk configurations where intensity or phase was measured [74–76]. In which concerns optical fibre based SPR, for which miniaturization is possible, the results obtained so far are similar to the ones demonstrated by other configurations based on LPFGs with thin films. The interaction of a mode guided by the fibre and the free electrons of a metallic film, deposited over the cladding, results in coupling to a surface plasmon wave that originates from a resonance dip in the transmission spectrum [77, 78]. For high sensitivity a single mode fibre is tapered in order to allow the interaction of the core mode with the thin film [79]. This approach has an added advantage of allowing the sensor to work in the 1.55 *μ*m wavelength range (Figure 16). Alternatively, under certain constrains (grating period and length), LPFG-assisted SPR can be employed [80–82]. This approach does not compromise the fibre integrity but limits the use of the sensor to the visible or near-infrared range (Figure 17).

**Figure 18:**
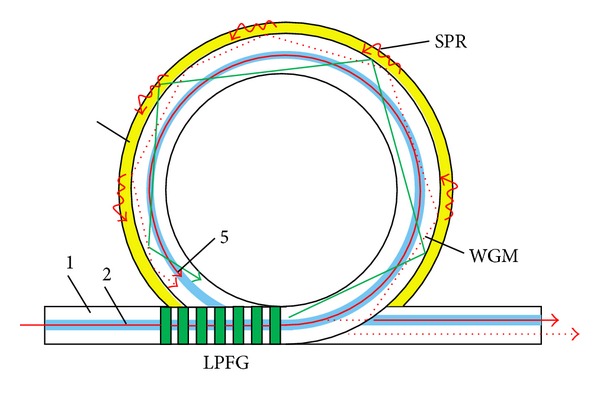
High resolution refractometric sensor comprising a LPFG, a WGM, and SPR (adapted from [93]).

When the film consists of metallic nanoparticles, the incident radiation interacts with the conduction electrons confined to the nanoparticles and is absorbed and scattered giving rise to localized SPR (LSPR) [83–85]. The resonance wavelength is strongly dependent on the particles size, shape, composition, and the dielectric properties of the surrounding medium. Although SPR is more sensitive, the response of LSPR sensors is more linear and its sensitivity can be tuned since it depends on the dimensions of the nanoparticles. A thin film surrounded by two media of close refractive index gives rise to the so-called long range SPR (LR-SPR), that is, a strong interaction on both sides of the metallic film that leads to a higher sensitivity (despite the larger FWHM) and low loss (for films with thickness lower than 25 nm) [86, 87]. Moreover, it allows coupling assisted by LPFGs with periods of hundreds of micrometers at a resonance wavelength of 1550 nm [88]. Recently, it was found that the deposition of a high refractive index silicon coating (doped with phosphorus), with 5 to 10 nm thickness, over the metallic layer increases considerably the sensitivity of the SPR sensor besides protecting the silver film from oxidation [89, 90]. Thus SPR with silicon coatings has been applied to the measurement of urea and pH [91, 92]. These innovative configurations involving SPR and LPFGs can be further extended to other devices such as nanotapers and WGM pushing forward the sensors sensitivity [93].  Figure 18 shows the possible inscription of a LPFG to assist coupling to a WGM avoiding the requirement of using small curvature radius that may damage the sensor. Refractive index resolutions exceeding 10^−8^ may be achieved by this configuration. Similar results are expected for optical fibre SPR based on phase measurements [94].

## 6. Conclusions

Sensors and, in particular, refractometric sensors have a key role in this smart era. Long period fibre gratings are intrinsically sensitive to the surrounding refractive index and have already demonstrated their potential for the implementation of this kind of sensors. The fabrication of highly sensitive sensors requires the inscription of LPFGs in the turning points. However, this procedure must be combined with the deposition of thin films over the grating, with the proper refractive index and thickness, in order to optimize the sensitivity to the measurement of refractive indices far from that of the cladding. Further increase in the sensitivity demands for the association of different techniques that lead to a higher interaction of the evanescent field with the parameter to be measured. Therefore, a reduction of the fibre cladding and/or a combination of LPFGs with SPR and/or WGMs may be the path for the development of ultrahigh sensitive refractometric sensors (exceeding resolutions of 10^−8^). As a final remark, it should be stressed that to make commercial devices available, besides the high resolution and the multiparameter measurement (namely, refractive index and temperature) exhibited by some sensor configurations, minor product engineering is still required in order to prevent cross-sensitivity of the sensor head to other physical parameters, such as strain and bending.
